# Supplementary material for: Social media-based Health Education plus Exercise Program (SHEEP) to improve muscle function among young-old adults with possible sarcopenia in the community: A feasibility study protocol
Source: PLoS One. 2025 Jan 3;20(1):e0303481. doi: 10.1371/journal.pone.0303481 (PMC11698421; doi:10.1371/journal.pone.0303481)
Supplement: S2 File — (PDF) [file pone.0303481.s002.pdf]

P11-12 Table 1

Figure**.** Example template of recommended content for the schedule of enrolment, interventions, and assessments.*

	STUDY PERIOD							
	**Enrolment**	**Allocation**	**Post-allocation**					**Close-out**
**TIMEPOINT****	***-t_1***	**0**	***t_1***	***t_2***	***t_3***	***t_4***	***etc.***	***t_x***
**ENROLMENT:**								
**Eligibility screen**	X							
**Informed consent**	X							
[List other procedures]	X							
**Allocation**		X						
**INTERVENTIONS:**								
[Intervention A]			◆—	—	—◆			
[Intervention B]			X		X			
[List other study groups]			◆—	—	—	—◆		
**ASSESSMENTS:**								
[List baseline variables]	X	X						
[List outcome variables]				X		X	etc.	X
[List other data variables]			X	X	X	X	etc.	X

*Recommended content can be displayed using various schematic formats. See SPIRIT 2013 Explanation and Elaboration for examples from protocols.

**List specific timepoints in this row.
